# Intertextual Reimagining of Desdemona: Feminist Revision, Postcolonial Memory, and Shakespearean Adaptation

**DOI:** 10.12688/f1000research.190656.1

**Published:** 2026-09-26

**Authors:** Majed Al-Anzy, Ayman Elhalafawy, Eman Darwish, Mohammed Abou Adel, Ahmad Al Mahamed, Samir Khalifa

**Affiliations:** 1 Northern Borders University, Arar, Saudi Arabia; 2 Kafrelsheikh University Faculty of Arts, Kafr El-Shaikh, Gharbia Governorate, Egypt; 3 United Arab Emirates University, Al Ain, Abu Dhabi, United Arab Emirates; 4 Abu Dhabi University, Abu Dhabi, United Arab Emirates; 5 North Private College of Nursing, Arar, Saudi Arabia

**Keywords:** Adaptation; Desdemona; Feminist revision; Intertextuality; Postcolonial feminism; Shakespeare

## Abstract

**Background:**

In an age increasingly concerned with gender justice, racial memory, and the decolonization of literary canons, Shakespeare’s Desdemona remains a critical site for rethinking how women’s voices are silenced, revised, and restored across cultures and historical moments. This study examines the intertextual reimagining of Desdemona in William Shakespeare’s *Othello*, Toni Morrison’s *Desdemona*, and Paula Vogel’s *Desdemona: A Play About a Handkerchief*, arguing that these works should not be read as isolated dramatic texts but as a tri-textual field of feminist revision and postcolonial critique. Addressing a gap in previous scholarship, which has largely treated Morrison’s and Vogel’s adaptations separately, the article offers a theoretically grounded comparative reading that brings the three plays into dialogue.

**Methods:**

Methodologically, the study employs qualitative intertextual analysis and close reading, drawing on Julia Kristeva’s intertextuality, Gérard Genette’s hypotext/hypertext model, Adrienne Rich’s feminist re-vision, Linda Hutcheon’s theory of adaptation, and Chandra Talpade Mohanty’s postcolonial feminism. It shows how Morrison and Vogel transform Desdemona from a passive, silenced figure within Shakespearean tragedy into a complex site of agency, resistance, memory, and feminist critique.

**Results:**

The findings reveal that Morrison restores Desdemona’s voice through purgatorial self-knowledge, Barbary’s recovered Black female memory, and cross-racial solidarity, whereas Vogel uses parody, class consciousness, sexual agency, and chamber-space disruption to dismantle patriarchy.

**Conclusions:**

This study has re-framed the problem of Desdemona’s representation not as a question of character revision alone, but as a broader inquiry into how canonical drama can be reread, contested, and politically reactivated through feminist and postcolonial intertextuality.

## Introduction

The significance of this study lies in its ability to redirect critical inquiry from individual representation to the broader issue of intertextual transformation across texts and cultures. Its originality resides in its tri-textual approach, which brings together Shakespeare’s *Othello (2025)*, Toni Morrison’s *Desdemona (2012)*, and Paula Vogel’s *Desdemona: A Play About a Handkerchief* as a single dialogic field through which Desdemona is reconfigured from an emblem of silence and obedience into a site of agency, memory, and feminist resistance. The study’s distinctiveness is further reinforced by its comparison of two divergent revisionary strategies: Morrison’s reparative recovery of Black female voice through memory and cross-racial solidarity, and Vogel’s parodic dismantling of patriarchal structures through the body, class, and theatrical space. Accordingly, the value of this research extends to adaptation studies, postcolonial feminism, and the decolonization of the literary canon, positioning it as a timely scholarly contribution with clear relevance to global critical debates ( Ayrim et al., 2026; Adel et al., 2025; Alenzi et al., 2026).

However, remarkable and creative texts come out from between fundamental literary production like those of Shakespeare’s. The handkerchief, being a theatrical prop, vibrantly represents a complicated and unsettled story of objects’ sentimental value, cross-cultural interaction, and a symbol that the original plot employs but does not fully clarify its history, as shown in Desdemona’s words: “Sure, there’s some wonder in this handkerchief.” The short, troubling mentions of Desdemona’s maid who raised her and whose name is not mentioned, but what is mentioned is that she fell in love and that it killed her as she died singing the Willow Song. This serves as a glimpse of the refusal to shed light on the black female experience. Additionally, Desdemona’s desires, thoughts, interior life, and ability to resist or comply are always subjected and tailored to fit the requirements of a tragic heroine. These missing pieces, surrounded by suppression and silence, provoke postcolonial and feminist writers, handing them a radical source for intertextual revision.

Revolutionary intertextual revisions have been produced in the late twentieth and early twenty-first centuries by feminist authors Paula Vogel and Toni Morrison: Vogel’s *Desdemona: A Play About a Handkerchief* (1993), a chamber comedy set in Cyprus, and Morrison’s *Desdemona* (2011), a collaboration with director Peter Sellars and musician Rokia Traoré, revisioning a purgatorial afterlife. Both plays acquire their critical significance by revisioning the original text and taking advantage of untold stories to speak up and represent marginalized female characters such as Desdemona, who has been placed as the righteous yet ill-fated Venetian wife whose innocence ignites the tragedy as represented in Emilia’s words: “Nay, lay thee down and roar; For thou hast kill’d the sweetest innocent That e’er did lift up eye,” and especially Barbary, whose voice is never heard. These texts do not just adapt but engage in an active dialogue with Shakespeare’s original text, rewriting Desdemona by shedding light on her interior life, relational autonomy, and clear overthrow of patriarchal norms.

The research problem that lies in this paper is explicit and momentous. As a big number of literary works focus on *Othello* from a feminist and/or postcolonial point of view, most of them receive Vogel’s *Desdemona: A Play about a Handkerchief* and Morrison’s *Desdemona* as unrelated examples, examining each individually in relation to Shakespeare’s original text, overlooking the tri-textual analysis demanded and well deserved due to their shared intertextual literary production. This critical current in the direction of this separation has concealed to what degree these two literary woreks complete each other cooperatively, shedding light on a feminist bond where a foundational dedication is shared, which lies in reclaiming females’ voice, identity, and autonomy.

The main research idea in this paper is to explain how Vogel’s *Desdemona: A Play about a Handkerchief* and Morrison’s *Desdemona* intertextually revise Shakespeare’s Desdemona, and what perceptions unfold from their opposing ways of dialogue expansion, narrative reversal, and tonal transposition. The goal of this paper is to illustrate that both writers adopt a foundational alteration of Desdemona through close and theoretically based intertextual reading. From a passive silenced symbol to an interactive representation of feminist resistance and independence. She appears interpersonal and racially aware in Morrison’s play, while being rebellious and socially aware in Vogel’s, showing the limitations and likelihood of female solidarity across different races, social classes, and history. Switching from the Shakespearean experience to these two hypertexts, this paper argues that adopting the intertextual theory can serve as a feminist critical practice along with a decolonizing strategy.

## Theoretical framework

The theoretical architecture of this study is built on several theories from different academic disciplines. The theories that relate to the concept of intertextuality, such as those of Julia Kristeva and Gerard Genette, as well as the feminist revisioning of Adrienne Rich, the theory of adaptation of Linda Hutcheon, and the postcolonial feminist approach of Chandra Mohanty, will be used in the investigation of these texts.

Kristeva’s theories of intertextuality, outlined in her text “Word, Dialogue and Novel” in 1980, state that every text contains another text within it, as she states: “Any text is constructed as a mosaic of quotations; any text is the absorption and transformation of another” ( Kristeva, 1980, p. 66). More specifically, she utilizes Bakhtin’s theory of dialogism to state that every text is created through the interaction of multiple texts and their ideas within the text being discussed. Furthermore, she states that there is no such thing as an original text with an original meaning; the meaning of a text is created through its interactions with other texts. Consequently, it is possible to understand the motivations of authors of texts like Morrison’s and Vogel’s hypertexts simply by recognizing that the text of Shakespeare’s Othello is itself created through the absorption and transformation of other texts, such as Cinthio’s *Hecatommithi* (1565), upon which Shakespeare’s *Othello* was based.

Gerard Genette’s *Palimpsests: Literature in the Second Degree* (1982; English translation 1997) provides a more formally oriented complement to the philosophically oriented work of Kristeva. Genette’s distinction between the hypotext and the hypertext, as he explains: “By hypertextuality I mean any relationship uniting a text B (which I shall call the hypertext) to an earlier text A (I shall, of course, call it the hypotext), upon which it is grafted in a manner that is not that of a commentary” ( Genette, 1997, p. 5), provides a means of discussing the relationship between *Othello* and its feminist revisions. More specifically, Genette’s discussion of the different types of hypertextual relationships (including categories such as parody, travesty, pastiche and transposition) enables an understanding of the different strategies that have been employed by both Morrison and Vogel in their revisions of Othello. Morrison’s approach to Desdemona can be categorized as using “serious transposition,” whereas Vogel’s approach falls more within the category of “travesty.”

Adrienne Rich’s influential essay “When We Dead Awaken: Writing as Re-Vision” (1972) introduces the concept of re-visioning as one of the essential components of the writings of female authors and in literature as a whole, as he says: “Re-vision – the act of looking back, of seeing with fresh eyes, of entering an old text from a new critical direction – is for us more than a chapter in cultural history: it is an act of survival” ( Rich, 1972, p. 18). For Rich, “re-visioning” involves viewing the literary pattern created by men and patriarchal society with what she refers to as “fresh eyes” and from a critical point of view. The concept of re-visioning functions as an “act of survival” for female writers who have had to reject the expectations and constructs of society regarding the roles of and expectations imposed on them. Both Vogel and Morrison engage with the topic of Desdemona in a way that expresses their feminist re-visioning and at the same time respects the character from Shakespeare’s play.

Canadian scholar Linda Hutcheon’s text *A Theory of Adaptation* (2013) is one of the more influential theories regarding the concept of adaptation in film and other media, as she says: “Multiple versions exist laterally, not vertically” ( Hutcheon, 2013, p. 169). Adaptations are not works that attempt to remain faithful to the content of the source texts, Hutcheon argues; they are works that creatively utilize those texts, those concepts, and their histories to create new forms of media with new meanings and implications - meanings that are based both in the content of the source text but in the history and culture of the adapting artists as well.

Chandra Talpade Mohanty’s work in both *Under Western Eyes* (1984) and “Under Western Eyes” Revisited: Feminist Solidarity (2003) provides a critical framework for understanding how many “feminist” analyses of literature, film, and other media ignore the differences within the category of “women” itself. In Mohanty’s work, any attempt to form cross-cultural connections within the “feminist” movement is inherently problematic if those two cultures are not experiencing the same challenges and oppressions - for instance, the different ways in which the oppression of slavery and colonialism has differently impacted women of different cultures and backgrounds, as she says: “The assumption of women as an already constituted, coherent group with identical interests and desires, regardless of class, ethnic or racial location or contradictions, implies a notion of gender or sexual difference or even patriarchy… which can be applied universally and cross-culturally” ( Mohanty, 1984, pp. 336–7). Thus, Mohanty’s arguments help to reveal the tricky aspects of both Morrison’s and Vogel’s works. Both plays are full of questions about solidarity and differences: How can women of different races and classes bond together against the oppression being posed upon them? Are there forms of complicity in the abuse women face that need to be addressed before solidarity becomes real?

The consolidated theoretical aspects dedicated in this paper, lying in Kristeva’s intertextuality, Rich’s feminist revisioning, Hutcheon’s adaptation theory, and Mohanty’s postcolonial feminism, add up to a reasonable and politically valuable analytical point of view. Their approaches combined allow the research to double play on a detailed level of textual analysis by taking a detailed look at specific scenes and speeches, and a wider look at the cultural analysis, displaying each writer’s approach and philosophy.

## Literature review

There has been a considerable amount of scholarship on the topic of feminist and postcolonial adaptations of Othello over the past thirty years. More specifically, there has been a critical discussion of the theme of adaptation as a form of feminist protest, analyses of Vogel’s *Desdemona: A Play About a Handkerchief*, and discussions of Morrison’s *Desdemona* in the context of Black feminist and postcolonial theory. However, a gap exists within this scholarship in that none of the studies have attempted to conduct a tri-textual analysis of Shakespeare’s *Othello*, Morrison’s *Desdemona*, and Vogel’s play.

One of the clusters of feminist adaptations of Othello that has been discussed in scholarly articles is the concept of adaptation as a form of feminist protest. Elizabeth Gruber (2023) discusses how the adaptation of stories by authors other than Shakespeare can be considered a form of protest and resistance performed by feminists (p.1). Another scholarly article by Neslihan Köroğlu, where she says: Ania Loomba (1989) highlights the interconnectedness of the formation of ‘the other’ for women and black people, remarking “each hierarchical structure of domination is analogous and linked with the other” (pp. 1–2).

The analogy Loomba discusses has long been ignored by mainstream feminism, which has failed to address the struggles of Black women while centering its arguments on the oppression of white women. A second cluster of scholarship on the topic of feminist adaptations of Othello focuses specifically on Vogel’s Desdemona. While D.G.J. Veldhuijzen’s “An Analysis of Paula Vogel and Toni Morrison’s Feminist Play Appropriations of Othello” (2016) is one of the few existing studies that compares both Vogel and Morrison, it does so primarily by distinguishing between the two authors’ different understandings of feminism and the role of difference within it. Vogel’s work has been the focus of a few studies that aim to provide feminist analyses of the play.

Milena Kaličanin ’s “Paula Vogel’s Desdemona: A Feminist Reading” (2019) examines the sexual explicitness and domestic focus of Vogel’s play. Her analysis of the play frames Vogel’s work as a form of comedy that contrasts with the tragedy of Shakespeare’s play. A. Mohamed’s “Reimagining Othello’s Wife” (2024) analyzes Vogel’s use of intersectionality within the play. While the play shares the experience of oppression between all three main female characters, it also explores the differences in their social and economic classes. The third of the three main themes that have been explored regarding Vogel’s Desdemona is the more theoretically sophisticated analysis of Morrison’s play. J.E. Carney’s “Being Born a Girl: Toni Morrison’s Desdemona” (2024) investigates the character of Desdemona’s girlhood and her relationship with Barbary. Morrison’s depiction of their relationship is presented as a means of reclaiming the erased history of Black women within the Shakespearean tradition.

 Erickson’s “Late Has No Meaning Here: Imagining a Second Chance in Toni Morrison’s Desdemona” (2013) studies Morrison’s descriptions of the purgatorial afterlife of the women in the play. Through these descriptions, Morrison provides the characters with a means of gaining knowledge of themselves and their actions throughout their lives. The most recent and comprehensive study of Toni Morrison’s Desdemona is that by Bayardo (2025) entitled “Harmony and Rupture in Desdemona: Toni Morrison’s Remaking of Shakespeare’s Othello”. The study analyzes the relationship between harmony and rupture within the play as a means of understanding the relationship that Morrison both respects and critiques in Shakespeare’s Othello. Following this entry in the field of Morrison’s Desdemona, Maria Valentini’s publication of “Toni Morrison’s Desdemona as an Expansion to the Interpretation of Othello” (2024) situates Morrison’s adaptation within the context of Morrison’s relationship to African American literature.

Other scholarship has increasingly adopted a new feminist, intersectional, post-colonial, and performance-based critical approach to literary and dramatic works that can help rethink female characters like Desdemona. Alenezi et al. (2026) disrupt the conventional feminine stereotypes by discussing the social and class constraints affecting women’s lives and highlighting the impact of patriarchal structures on women’s identities and agency. Similarly, Alhourani et al. (2025a) and Elhalafaway et al. (2025) use the lenses of trauma and intersectionality to explore how gender oppression intersects with other forms of marginalization, providing a useful lens for thinking about female characters impacted by multiple social and cultural forces. The postcolonial approach of Alhourani et al. (2025b) investigates the decolonization of the stage, and they show how modern drama may begin to disrupt the majority narratives in culture and reveal postcolonial discourses.

In the same way, Alrefai et al. (2026a) and Khalifa et al. (2025) focus on intersectionality and marginalization in The Sympathizer, exploring the intersections between identity, cultural difference, and social marginalization. In addition, Alrefai et al. (2026b) and Abou Adel et al. (2025) explore how the dialogic tension in Caryl Churchill’s Seven Jewish Children allows for a range of voices, ideologies, and alternative viewpoints to be accommodated within dramatic texts, using a Bakhtinian approach. Together, these studies demonstrate the value of feminist, intersectional, postcolonial, and dialogic approaches to the reinterpretation of literary and dramatic narratives, and constitute a solid base for looking at the intertextual reimagining of Desdemona as a space of feminist revision, postcolonial memory, and Shakespearean adaptation.

Despite the number and the richness of these scholarly publications on the topic of Morrison and Traore’s adaptations of Desdemona, the gap in the knowledge identified at the outset of this review is still present and substantial. While Veldhuijzen’s publication was a pioneering study on the two authors to be discussed in this study, it was limited to an undergraduate research project. Additionally, other publications that have investigated the adaptations of Desdemona by Morrison and Traore have not made tri-textual analysis a central focus, as the present study does. By bringing Shakespeare, Morrison, and Vogel into conversation with one another within a theoretically grounded framework, this paper helps to fill the gap in the available scholarship on Desdemona and her transformation in particular in a way that no study of any of these authors’ work separately presents.

## Methodology

This study adopts a qualitative intertextual methodology as the most appropriate critical approach for examining the reimagining of Desdemona across three interconnected dramatic texts: Shakespeare’s *Othello*, Toni Morrison’s *Desdemona*, and Paula Vogel’s *Desdemona: A Play About a Handkerchief.* The analysis is informed by Julia Kristeva’s theory of intertextuality in “Word, Dialogue and Novel” (1980), Gérard Genette’s distinction between hypotext and hypertext in *Palimpsests* (1982/1997), Adrienne Rich’s concept of feminist re-vision in “When We Dead Awaken” (1972), Linda Hutcheon’s theory of adaptation in *A Theory of Adaptation* (2013), and Chandra Talpade Mohanty’s postcolonial feminism in “Under Western Eyes” (1984) and “Under Western Eyes Revisited” (2003). This methodology is selected because it reveals the aesthetic, ideological, and political transformations through which Desdemona moves from silence and submission to voice, agency, and resistance. Procedurally, the study first maps the intertextual motifs linking the three plays, including the handkerchief, the Willow Song, Barbary, and Cyprus. It then conducts a comparative thematic analysis of female interiority, gendered bonds, race, class, patriarchal authority, and textual subversion.

To clarify the analytical movement of this study, Figure 1 visualizes the intertextual transformation of Desdemona from Shakespeare’s hypotext to Morrison’s and Vogel’s feminist hypertexts. The figure summarizes how intertextuality operates as both a method of close reading and a feminist decolonizing strategy.

**Figure 1.**
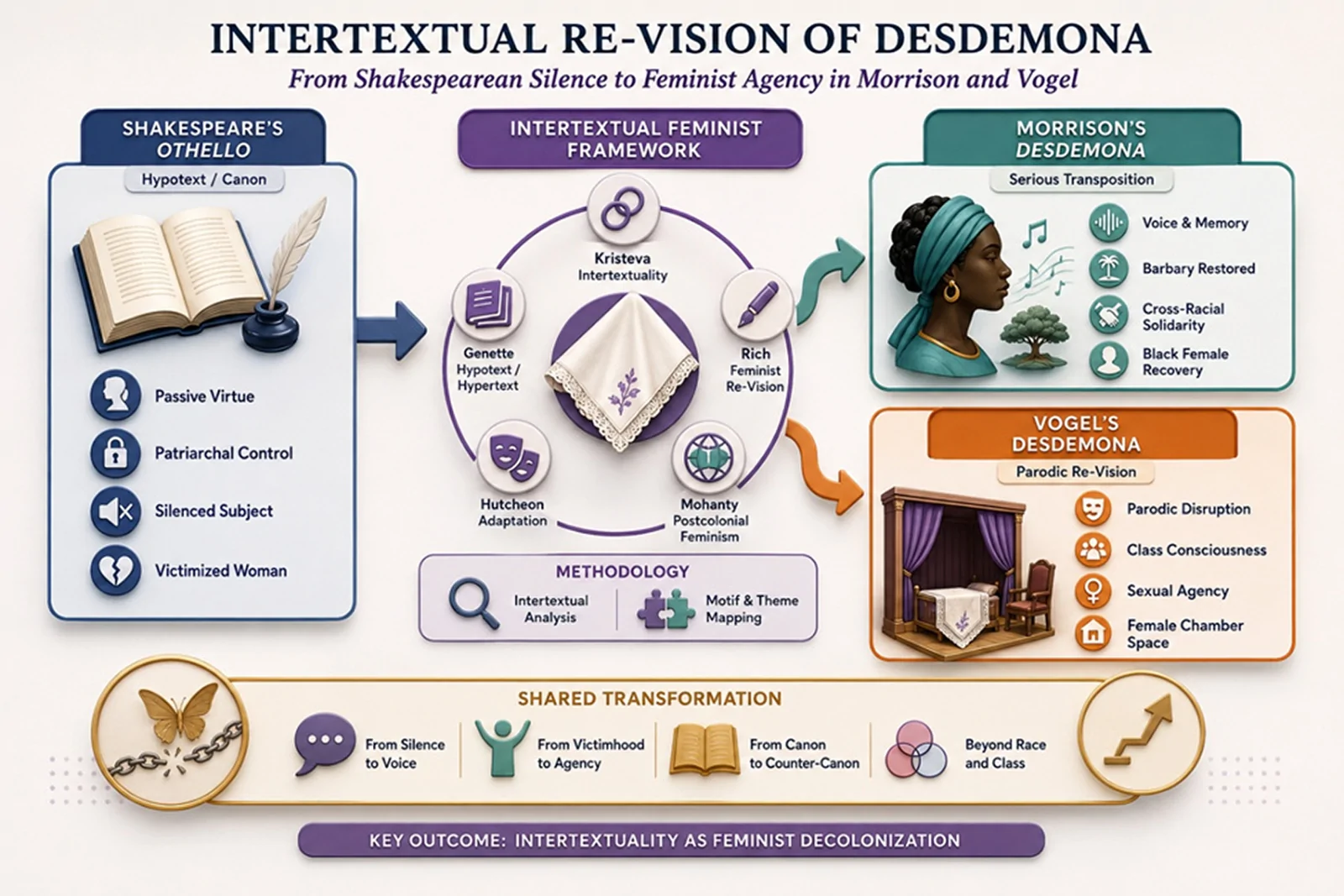
Intertextual re-vision of desdemona across shakespeare, morrison, and vogel. The figure illustrates the transformation of Desdemona from a silenced character in Shakespeare’s Othello into a figure of feminist agency in the adaptations of Toni Morrison and Paula Vogel.

## Discussion and findings

Shakespeare’s Othello has always been a fundamental reference for centuries all over the literary globe for themes of jealousy, gender, identity, and the destructive effect of domestic violence. Desdemona’s fidelity, beauty, and innocence as a Venetian noblewoman contribute to her downfall by the end of the play ( Khater, 2022). She is situated as the symbol of virtue and a traditional wife in a society that requires obedience and control over women for them to fall under patriarchal law, as she says: “I do perceive here a divided duty” (Shakespeare, p. 15). Desdemona says this line upon recognizing that she is caught between her father and her husband, as women were to be obedient to the man in their life. Here, she is transitioning from being loyal to her father to being loyal to her husband. Furthermore, her quote demonstrates her awareness of, and obedience to, the societal expectation to be loyal to her husband. This later leads to her tragic fall in the play due to her unwillingness to defend herself against her husband. Additionally, Othello murdering Desdemona with his own hands without even a second thought has always been viewed as the result of destructive jealousy, misplaced blind trust, and disastrous possessiveness, as foreshadowed in Iago’s words: “Oh, beware, my lord, of jealousy! It is the green-eyed monster which doth mock the meat it feeds on” (p. 48). And in Othello’s words: “Be thus when thou art dead, and I will kill thee, and love thee after” (p. 91).

In constructing the life of Desdemona, Shakespeare displays his typical attributes of women who are considered to be virtuous within the plays of the early modern era. From her introduction to the play, when she must explain to her father Brabantio the reasons for which she has chosen Othello as her partner despite his status and social position, to her description of her desire to view Othello’s mind in order to understand the man and his adventures, Desdemona portrays herself as a woman who is willing to submit to the decisions of her partner and who derives her feelings of love for Othello from her observation of him performing heroic actions that exclude her from the action itself. Beyond these descriptions of Desdemona within the play, she continues to exhibit her passive nature throughout the play; she often finds herself the victim of Othello’s abusive behavior toward her, despite her apparent innocence in the behaviors which he directs toward her.

The innocence of Desdemona is contrasted with the viciousness of Othello’s actions against her, highlighting both the helplessness of Desdemona and the tragedy of her eventual death at the hands of the man whose love she endured. Desdemona’s submissive response to Othello’s violent murder of her when she says: “Nobody; I myself. Commend me to my kind lord,” (p. 247) has been considered by numerous feminist critics to be the ultimate display of passivity on the part of women within Shakespeare’s play, effectively removing from Othello any responsibility for the crime that he committed against his wife.

However, the play does contain a number of other elements and motifs that go beyond and into the dominant construction of the play. Beyond the relationship that Desdemona had with Barbary during her girlhood, the play also explores other elements that suggest a history of the women within the play apart from that which is featured within the story itself. For instance, not only was Barbary mentioned as being in love with a man who became mad and abandoned her, leading to her death singing the Willow Song, but Desdemona also sang the song in her sleep prior to her death, which implies a relationship between the two women to the audience. Furthermore, elements like the handkerchief also contain a suppressed history within the play, as Desdemona says: “Sure, there’s some wonder in this handkerchief” (Shakespeare, 1603–04, p. 61). It has been revealed to have been given to Othello by his mother, who was enchanted by a sibyl; elements related to this object are discussed by both Vogel and Morrison within their analyses of the play.

Toni Morrison achieves her most fundamental intertextual transformation of Shakespeare’s play through the radical reimagining of time and space that her purgatorial setting provides for her protagonist. By transporting the character of Desdemona to a purgatorial afterlife that exists outside of time, history, and social hierarchy, Morrison creates space for Desdemona to gain a retrospective form of self-knowledge that would have been impossible within the parameters of Shakespeare’s play. Outside of the constraints of her need to show the characteristics of a virtuous woman for a male audience, Desdemona is released to speak honestly to her audience about her life, her motivations, her failures, and her relationship to the societal restraints of her existence.

The purgatorial setting allows full existence for Barbary, a character that existed only as a name within Shakespeare’s play. In Morrison’s play, Barbary is given a complete and complex dramatic portrayal of her character. A voice, a history, and a perspective that exists in the cradle of West African traditions, as well as the relationship that she has with Desdemona. While Morrison’s Desdemona expresses her love for Barbary and her disappointment at the separation of the two women as a result of social hierarchy, Barbary expresses her displeasure at the thought of such a relationship between the two women and states: “So you don’t know me. Have never known me” (p. 9). Through these words spoken by Barbary, Desdemona is made aware of the limitations of Barbary’s perception of the two women during their lives.

In the same way that Morrison reimagined the role of the characters in the play, she also reimagined the role of Willow Song. Rather than the anticipation of Desdemona’s death as interpreted by Shakespeare, Morrison’s version of the song includes the memories of Barbary of her cultural traditions, her world, and the freedom that the two women felt in singing the song together. This symbol forms a mosaic of various references: to Shakespeare’s mention of the Willow Song in the play, to Morrison’s own references to West African music through Rokia Traore’s compositions, and to the suppressed knowledge of the origin of the song referenced by Shakespeare. The original play by Shakespeare is thus both remembered and transformed within Morrison’s text.

This intertextual element extends beyond Desdemona’s relationship with Barbary to her marriage to Othello and her place in the social structures around him. While Desdemona accepts the marriage and the violence from Othello within the original play, Morrison’s Desdemona can finally step outside of this acceptance. She states: “My life was shaped by my own choices and it was mine” (p. 1). Though this acknowledges the use of self-deception, it is also true that a life shaped by seemingly “own” choices within a social world structured around oppressive ideologies of gender and race was both true of Morrison’s Desdemona and a necessary act of resistance.

The fact that Morrison’s project was created in collaboration with Peter Sellars and Rokia Traore is itself a form of intertextuality, as the process of creating the production was a means of putting on the intercultural solidarity that was represented within the play’s content. Traore’s music for the play utilized languages and musical traditions from West Africa, specifically those associated with the griot class of musicians and storytellers, which was a cultural and musical element that was impossible for Shakespeare to have conceived of during his time. Thus, the production was polyphonic both in its literal and metaphorical meanings, as its inclusion of different languages, musical traditions, and various cultures helped to illuminate each other to the audience.

In absolute contrast to Morrison’s production of Desdemona, Paula Vogel’s Desdemona: A Play About a Handkerchief takes place in a completely different aesthetic. Vogel’s play is characterized as irreverent, comedic, bawdy, and altogether anti-Shakespearean in its structure and content. Vogel utilizes parody to create what is essentially a radical feminist deconstruction of the concepts that underlay Shakespeare’s Desdemona. While Morrison sought to supplement and repair Shakespeare’s silence regarding the characters he marginalized, Vogel attacked and dismantled the symbolic system that Shakespeare put together. Vogel’s setting of the play to the chamber in Cyprus, a location in which the women of the play reside during the scenes in which they do not appear in the play’s male-dominated spaces, completely removes the focus of this play from the focus of the hypotext. The political world of the play is moved completely off-stage, focusing instead on the setting that Vogel constructed as the center of this one.

Within the chamber of Cyprus, Vogel’s three characters - Desdemona, Emilia, and Bianca - are subject to parodic interpretations of their Shakespearean characters. While Desdemona in the original play by Shakespeare is the innocent and faithful wife of her husband Othello, as Emilia mourns her and says: “Nay, lay thee down and roar; For thou hast kill’d the sweetest innocent That e’er did lift up eye,” (Shakespeare, p. 98), in Vogel’s interpretation, she is an unfaithful aristocrat who has had affairs with several soldiers in the Cypriot soldiers. Vogel’s alteration of Desdemona as a character to be this different and unapologetic for it is a means of rejecting the idea of female virtue correlating to female passivity in the play.

The handkerchief that is central to the play and Iago’s plot is replaced by Vogel with a mundane item that has been passed from man to woman several times throughout the play, demonstrating the abandoned nature of the handkerchief and, therefore, utterly diminishing the notion of the handkerchief as a symbol of virtue in the original play by Shakespeare. This aspect of the Genettian transposition of Shakespeare’s drama is actually only one aspect of Vogel’s general strategy of showing the genre and the practices of Shakespeare’s tragedies.

Class, for instance, is one of the elements of the play that Shakespeare seeks to obscure in his tragedy. While Desdemona and Emilia are represented as the mistress and her servant in the household, their relationship is one of mutual dependence, affection, and resentment. Furthermore, Emilia’s perspective as the Irish servant to Desdemona provides both a sympathetic yet critical viewpoint of Desdemona’s romances and multiple relationships with men; she understands the privileges associated with Desdemona’s wealth and position in society. In contrast, Bianca, the Cypriot prostitute in the play, is the most marginalized of the three women in *Othello.*

Vogel’s interpretation of this character is of a strong, pragmatic woman who is used to the brutalities of prostitution and has no illusions regarding the romantic possibilities for Desdemona or the servitude of Emilia to their household. By deploying such distinctly colloquial, bawdy, and medieval elements within her hypertext, Vogel successfully employs Bakhtin’s notion of the carnivalesque to downplay the distinctions between different categories of Shakespeare’s play. Yet Vogel’s deployment of such elements also simultaneously highlights the ways in which the lives of the three female characters in the hypertext are still within significant constraints, and in the limitations of their relationships with one another.

Through reading both Toni Morrison’s and Vogel’s hypertexts alongside Shakespeare’s originating play, it becomes possible to recognize the ways in which both authors exhibit similarities in the strategies that they employed within their contributions to the dialogue concerning Shakespeare’s work. Yet, it is also possible to recognize the differences between those authors, as well. Both of these feminist authors work to write back to Shakespeare from the perspective of the female characters of the play, excluding the male characters and their destructive behaviors of jealousy and violence towards the females of the play. Both mend and amplify the female bonds that exist within the play by Shakespeare. Furthermore, both of these interpretations work to transform the relationship between the female characters and the symbolic elements of the play in a way that highlights the function of the symbolism in it, as well as suggests alternative functions for those symbols: functions that are feminist in their concept.

One of the main differences between Morrison and Vogel is in their relationships to the concept of solidarity between females of difference. Morrison focuses upon the concept of solidarity between females of different races to the extent that she must examine the ways in which solidarity between the different types of women does not exist due to racial differences between those females, while Vogel utilizes the comedies and parodies within the play to expose the class differences between the female characters within patriarchal society, without focusing upon any attempts at solidarity between those female characters of the play. The contrasting intertextual strategies that Morrison and Vogel employ within their plays reflect the divergent themes that their plays explore. Morrison’s play assumes the relevance of Shakespeare’s text and the need to supplement it with her own interpretations of the erased and silent characters. Vogel’s play, in contrast, takes a more skeptical view of Shakespeare’s text and its intentions.

The character of Barbary illustrates these different themes and their approaches to Shakespeare’s text. Morrison’s inclusion of Barbary represents the insertion of a character that reflects the play’s focus on the experiences of Black women. Vogel’s play, however, omits the character of Barbary altogether. This contrast indicates the different priorities of the two playwrights. While Morrison is focused on creating the recovery of the Black female as the basis of her feminism, Vogel is focused on the impact of class and gender across races of women.

The discussion that preceded leads to an outcome that comprehensively answers the research questions. The answer to the question of in what ways Morrison’s *Desdemona* and Vogel’s *Desdemona: A Play About a Handkerchief* intertextually reimagine Shakespeare’s Desdemona reveals that while both playwrights utilize some similarities in their approaches to writing the character of Desdemona, they also differ in some of the strategies that they employ in their plays.

Both playwrights work to transform the passive and voiceless character of Desdemona in Shakespeare’s play into a Desdemona who possesses a life and the ability to exhibit the type of active personality that was denied to her within Shakespeare’s play. Morrison creates such a transformation by adding the consciousness of the purgatorial Desdemona, while Vogel accomplishes such a transformation by creating a Desdemona that challenges the ideals of Shakespeare’s play through her social class.

The second key finding pertains to the nature and limits of female solidarity as each playwright constructs it within their plays. In Morrison’s play, the solidarities between women of different races are both the essence and the problem of the play. The relationship between Desdemona and Barbary represents the possibility of solidarities between women of different races, but also the limitation of such solidarities in history due to the existence of white privilege and the racial hierarchies that have existed between different racial groups. Similarly, solidarity between women of different classes is both possible and problematic in Vogel’s play; the solidarities that exist between Desdemona, Emilia, and Bianca are genuine but limited by the class differentials that exist between them.

The third key finding regarding both plays is that the use of intertextuality functions as a form of feminist decolonization within the plays. Both authors use intertextuality to bring the voices of women who have been silenced within Shakespeare’s plays to the center of the dramatic universes that they created. Through such a change, both authors make clear the ways in which the original play is incomplete and governed by ideologies that are not reflective of the diversity of the female experience; the reconstruction of such plays is, therefore, both a political and an ethical necessity.

## Conclusion

We conclude that the study has answered its central questions: Morrison and Vogel both transform Desdemona from a silenced emblem of obedience into a site of agency, critique, and counter-memory by bringing Shakespeare’s *Othello*, Toni Morrison’s *Desdemona*, and Paula Vogel’s *Desdemona: A Play About a Handkerchief* into a single comparative field, yet they do so through distinct aesthetic and ideological strategies. Morrison reconstructs Desdemona through afterlife dialogue, racial memory, Barbary’s restored voice, and the possibility—though not simplicity—of cross-racial female solidarity. Vogel, by contrast, uses parody, sexual frankness, class tension, and chamber-space disruption to expose the instability of patriarchal ideals embedded in Shakespeare’s tragedy.

The scientific contribution of this study lies in its tri-textual model of analysis. Rather than treating the two adaptations as separate responses to Shakespeare, it demonstrates how they illuminate one another as divergent feminist hypertexts: one reparative and memorial, the other parodic and demystifying. In doing so, the research contributes to wider debates on adaptation, feminist re-vision, decolonizing the canon, and the politics of recovering marginalized voices within global literary traditions.

The relevance of this inquiry extends beyond Shakespeare studies. In a contemporary world still shaped by gender violence, racial hierarchy, cultural memory, and struggles over canonical authority, these plays reveal how literature can interrogate inherited narratives and generate more inclusive forms of interpretation. Future research may extend this model to other *Othello* adaptations, performance-based comparisons, digital rewritings, and cross-cultural stagings, especially those that foreground race, class, music, embodiment, and non-Western modes of theatrical memory.

## Ethics approval

None of the authors of this article has studies involving human subjects.

## Disclosure of AI use

The authors used OpenAI’s ChatGPT (GPT-5.6) premium only to generate the study’s visual abstract ( Figure 1). The tool was not used in writing, analysis, interpretation, or formulation of the manuscript’s findings. All AI-assisted output was reviewed and approved by the authors.

## Disclosure statement

The authors declare that there are no competing interests.

## Data Availability

Data sharing is not applicable to this article as no data were created or analysed in this research.
