# Supplementary material for: Intertextual Reimagining of Desdemona: Feminist Revision, Postcolonial Memory, and Shakespearean Adaptation
Source: F1000Res. 2026 Oct 26;15:1604. Originally published 2026 Sep 26. [Version 2] doi: 10.12688/f1000research.190656.2 (PMC13662385; doi:10.12688/f1000research.190656.2)
Supplement: Peer review history

# Reviewer report: Reviewer response for version 2

**DOI:** 10.5256/f1000research.213048.r533866

**Published:** 2026-11-12

**Authors:** Nidal Al-Shamali

**Affiliations:** 1 Al-Balqa Applied University, Salt, Jordan

I have no further comments.

 I approve the research in its current form.

 Best regards.

Is the work clearly and accurately presented and does it cite the current literature?

Yes

If applicable, is the statistical analysis and its interpretation appropriate?

Not applicable

Are all the source data underlying the results available to ensure full reproducibility?

Yes

Is the study design appropriate and is the work technically sound?

Yes

Are the conclusions drawn adequately supported by the results?

Yes

Are sufficient details of methods and analysis provided to allow replication by others?

Yes

Reviewer Expertise:

His areas of expertise include Arabic literature and criticism, narratology, modern critical theories, discourse analysis, cultural criticism, comparative literature, gender representation, identity and otherness, Arabic heritage and classical literary criticism, Andalusian literature, and the application of generative artificial intelligence in language education.

I confirm that I have read this submission and believe that I have an appropriate level of expertise to confirm that it is of an acceptable scientific standard.

# Reviewer report: Reviewer response for version 1

**DOI:** 10.5256/f1000research.210434.r525155

**Published:** 2026-10-13

**Authors:** Baker Bani-Khair

**Affiliations:** 1 The Hashemite University, Az-Zarqa, Zarqa Governorate, Jordan

The authors in the paper titled, " Intertextual Re-imagining of Desdemona: Feminist Revision, Postcolonial Memory, and Shakespearean Adaptation" have successfully discussed the idea of woman representation, especially the classical woman voice that brings another stronger version of Desdemona in adapted works of Shakespeare's Othello, using various essential theoretical perspectives pertaining to Linda Hutcheon, Rich, and Mohanty. It is evidently clear that the authors followed a good writing style in both the organization and format of the paper and the textual analysis supported by selected scholarly citations. The authors have also taken good care of documentation and originality matter with careful attention to coherence and consistency throughout the paper. The paper's language is also excellent with good use of diction and clarity. The authors' contribution in the paper is also evident, and the research findings have definitely met the paper's objectives.

Is the work clearly and accurately presented and does it cite the current literature?

Yes

If applicable, is the statistical analysis and its interpretation appropriate?

Not applicable

Are all the source data underlying the results available to ensure full reproducibility?

Yes

Is the study design appropriate and is the work technically sound?

Yes

Are the conclusions drawn adequately supported by the results?

Yes

Are sufficient details of methods and analysis provided to allow replication by others?

Yes

Reviewer Expertise:

Gothic Literature

I confirm that I have read this submission and believe that I have an appropriate level of expertise to confirm that it is of an acceptable scientific standard.

# Reviewer report: Reviewer response for version 1

**DOI:** 10.5256/f1000research.210434.r525159

**Published:** 2026-10-08

**Authors:** Nidal Al-Shamali

**Affiliations:** 1 Al-Balqa Applied University, Salt, Jordan

**Suggested Revisions**

 The manuscript is well conceived, original in scope, and makes a valuable contribution to feminist adaptation studies, postcolonial criticism, and Shakespearean scholarship. The following minor revisions are recommended to further enhance its precision and presentation:

**Research gap and originality:** Refine the claim of novelty by distinguishing the article’s specific contribution—particularly its tri-textual comparative framework and its analysis of race, class, and revisionary strategies—from earlier comparative work on Morrison and Vogel.

**Methodological clarity:** Briefly specify the units of analysis (e.g., the handkerchief, the Willow Song, Barbary, and theatrical space), the comparative criteria, and the analytical steps linking textual evidence to interpretation. A concise comparative table may improve clarity.

**Theoretical coherence:** Clarify the distinct function of each theoretical framework. The relationship between *travesty* and *parody* in the discussion of Vogel’s play would benefit from fuller justification. If Bakhtin’s carnivalesque is retained, it should be introduced in the theoretical framework.

**Shakespearean interpretation:** Qualify broad characterizations of Desdemona as wholly passive and of Othello’s actions as unreflective. Brief engagement with alternative critical readings and closer analysis of selected scenes would strengthen the argument. The description of Barbary should also be corrected: she is identified as Desdemona’s mother’s maid in *Othello*.

**Textual evidence and citation:** Standardize the edition of *Othello* and, where possible, use act, scene, and line references alongside page numbers. Ensure that editions and page references for Morrison’s and Vogel’s texts are fully verifiable, and support historical or production-related claims with appropriate sources.

**Literature review:** Further develop the review as a critical dialogue with the most directly relevant scholarship. Reducing tangential references would sharpen the articulation of the research gap.

**Language, editing, and presentation:** A final professional language edit is recommended to correct minor grammatical and typographical inconsistencies, standardize titles and dates, verify bibliographic details, and refine the title and visual presentation of Figure 1.

**Final Recommendation**

**Approved.**

 The manuscript is suitable for indexing in its current form. The above suggestions are offered solely as constructive, non-mandatory refinements that may further strengthen the article’s clarity, methodological transparency, and scholarly presentation.

**Use of Artificial Intelligence**

 In the interest of academic integrity and transparency, we used artificial intelligence tools solely for language proofreading and editorial refinement of this review. No AI tool generated any part of the scholarly assessment, peer-review comments, or final recommendation. All observations and recommendations are based on the reviewer’s independent academic reading and professional judgment.

Is the work clearly and accurately presented and does it cite the current literature?

Yes

If applicable, is the statistical analysis and its interpretation appropriate?

Not applicable

Are all the source data underlying the results available to ensure full reproducibility?

Yes

Is the study design appropriate and is the work technically sound?

Yes

Are the conclusions drawn adequately supported by the results?

Yes

Are sufficient details of methods and analysis provided to allow replication by others?

Yes

Reviewer Expertise:

His areas of expertise include Arabic literature and criticism, narratology, modern critical theories, discourse analysis, cultural criticism, comparative literature, gender representation, identity and otherness, Arabic heritage and classical literary criticism, Andalusian literature, and the application of generative artificial intelligence in language education.

I confirm that I have read this submission and believe that I have an appropriate level of expertise to confirm that it is of an acceptable scientific standard.
